# The psychological impact of the COVID-19 crisis is higher among young Swiss men with a lower socioeconomic status: Evidence from a cohort study

**DOI:** 10.1371/journal.pone.0255050

**Published:** 2021-07-29

**Authors:** Simon Marmet, Matthias Wicki, Gerhard Gmel, Céline Gachoud, Jean-Bernard Daeppen, Nicolas Bertholet, Joseph Studer

**Affiliations:** 1 Addiction Medicine, Lausanne University Hospital and University of Lausanne, Lausanne, Switzerland; 2 Addiction Switzerland, Lausanne, Switzerland; 3 Centre for Addiction and Mental Health, Toronto, ON, Canada; 4 University of the West of England, Bristol, United Kingdom; University of Edinburgh, UNITED KINGDOM

## Abstract

**Aims:**

The present study aimed to investigate whether the psychological impact of the COVID-19 crisis varied with regards to young Swiss men’s pre-crisis level of education and socioeconomic status and to changes in their work situation due to it.

**Methods:**

A cohort of 2345 young Swiss men (from 21 out of 26 Swiss cantons; mean age = 29) completed survey-based assessments shortly before (April 2019 to February 2020) and early on during the COVID-19 crisis (May to June 2020). Outcomes measured were psychological outcomes before and during the COVID-19 crisis (depression, perceived stress and sleep quality), and the fear, isolation and psychological trauma induced by it. We investigated associations between these outcomes and their predictors: pre-crisis socioeconomic status (relative financial status, difficulty paying bills, level of education), changes in work situation during the crisis (job loss, partial unemployment, working from home, change in workload) and working in contact with potentially infected people, both inside and outside the healthcare sector. For outcomes measured before and during the crisis, the analyses were adjusted for their pre-crisis levels.

**Results:**

About 21% of participants changed their employment status (job loss, partial unemployment or lost money if self-employed) and more than 40% worked predominantly from home during the COVID-19 crisis. Participants with a lower relative socioeconomic status already before the crisis experienced a higher psychological impact due to the COVID-19 crisis, compared to participants with an average socioeconomic status (major depression (b = 0.12 [0.03, 0.22]), perceived stress (b = 0.15 [0.05, 0.25]), psychological trauma (b = 0.15 [0.04, 0.26]), fear (b = 0.20 [0.10, 0.30]) and isolation (b = 0.19 [0.08, 0.29])). A higher impact was also felt by participants who lost their job due to the COVID-19 crisis, the partially unemployed, those with an increased workload or those who worked mainly from home (e.g. depression b = 0.25 [0.16, 0.34] for those working 90%+ at home, compared to those not working at home).

**Conclusions:**

Even in a country like Switzerland, with relatively high social security benefits and universal healthcare, the COVID-19 crisis had a considerable psychological impact, especially among those with a lower socioeconomic status and those who experienced deteriorations in their work situation due to the COVID-19 crisis. Supporting these populations during the crisis may help to prevent an amplification of inequalities in mental health and social status. Such support could help to lower the overall impact of the crisis on the mental well-being of Switzerland’s population.

## Introduction

The coronavirus disease (COVID-19) pandemic caused by severe acute respiratory syndrome coronavirus 2 (SARS-COV-2) not only posed a direct risk to the physical health of people living in Switzerland and worldwide but also caused much psychological distress due to fears of what the infection might do to their health and that of others. In addition, official measures against the spread of COVID-19 caused economic uncertainty and, potentially, stress. Crises do not hit everyone equally. Instead, they often highlight and amplify pre-existing social and economic inequality [1]. The present study used data from a sample of young Swiss men (mean age 29; SD = 1.28 during the crisis) who participated in a long-term cohort study. One wave of surveying occurred shortly before the COVID-19 crisis, and participants were contacted again during the first wave of the COVID-19 crisis. This cohort study thus offered a unique possibility to investigate the impact of the COVID-19 crisis prospectively. The present study aimed to investigate whether the psychological impact of the COVID-19 crisis differed according to participants’ socioeconomic status (SES) and changes in their work situation.

At the beginning of March 2020, COVID-19 infections started to increase rapidly in Switzerland, and by 16 March there had been 3747 confirmed cases or 43.9 cases per 100,000 inhabitants [2]. On that day, the Swiss government announced drastic measures to halt the spread of the coronavirus (hereafter COVID measures). These included the closure of schools, restaurants, non-essential shops, tourism sites and other locations, as well as the introduction of social/physical distancing measures (2 meters between individuals and a maximum of five people per group) [3]. There were also recommendations to stay at home and work from home. These measures had a massive impact on the Swiss population’s daily lives and Switzerland’s economy: many jobs were endangered. Switzerland’s government took measures to reduce the pandemic’s impact on the workforce by offering employees the possibility of receiving partial unemployment benefits, in which a company could, instead of laying off employees, considerably reduce their working hours with most of the difference in salary being covered by the national unemployment insurance fund. To support the civil authorities, some young men were also called up to serve in their military or civil protection service units.

The COVID-19 crisis was expected to have a huge psychological impact [4, 5], and initial results confirmed this [6–9].A previous publication [7] on the sample of our study also reported that there was considerable psychological impact in our sample in form of psychological trauma, and fear (mainly for others) due to the COVID-19 crisis. Experiences from earlier crises [10], such as natural disasters (*e*.*g*. tsunamis) or pandemics (*e*.*g*. MERS), have also suggested that not everyone is equally affected by them [1, 11–13]. During a pandemic, vulnerable populations are more likely to catch the illness or be more strongly affected by it. They may also be more likely to feel the impact of the measures taken to contain the disease and their economic consequences [12, 14]. People with a lower SES form one of these vulnerable population groups, as they are disproportionally affected by physical [15] and mental health problems [16] in normal times, and these issues may be further exacerbated when crises occur [11, 17, 18]. They may also be more likely to lose their jobs or see a loss in income as they often work in sectors (*e*.*g*. restaurants, tourism) that are highly affected by crises [13]. Additionally, they may not have the financial resources and flexibility to counter a temporarily lower income, factors that may increase the psychological impact of a crisis for this group. However, empirical findings regarding associations between indicators of SES (income or education) and psychological impact of the COVID-19 crisis have been inconsistent. A review including studies until May 2020 identified lower socioeconomic status as a common risk factor (although not significant in all studies) between studies [19], while some later studies reported either no association [20, 21], or a higher impact in the group with above average income [22, 23], respectively middle income group [24].

Changes to working conditions due to the crisis, such as partial or full unemployment, loss of revenue and having to work primarily from home, may also have led to the COVID-19 crisis causing different psychological effects. For example, working from home may protect employees from infection with COVID-19 [1, 9], and two studies from Austria and Switzerland also identified the possibility to work from home as a protective factor the psychological impact of the COVID-19 crisis [9, 25], although in the second study this was only the case for one indicator (perceived stress measured with one question) and before adjustment for socioeconomic resources. However, working mostly from home may also lead to more social isolation and thus to increased stress. Furthermore, workers in direct contact with the public, for example, in supermarkets or hospitals, may be differently affected in their mental well-being, as they have a greater risk of contracting the virus [19, 26–30].

While there is a large body of literature describing the psychological impact of the COVID-19 crisis, there are still gaps in the literature about which subgroups are mostly affected by the crisis, and there are only relatively few longitudinal studies investigating this. Better knowledge of whether individuals with lower socioeconomic status or a difficult work situation during the crisis might suffer a greater psychological impact from the COVID-19 crisis is important for targeted interventions during the pandemic, but especially in its aftermath, as those most affected by the crisis will probably take longer to recover. This knowledge will also help healthcare professionals and policy-makers plan post-crisis healthcare needs and develop measures to counter the impact of future similar crises.

### Aims

The present work focuses on associations between COVID-19’s psychological impact and participants’ SES and work situation and aims to identify subgroups that suffer a higher psychological impact due tothe COVID-19 crisis. Specifically, the study’s first aim was to describe how the COVID-19 crisis affected the work situation of our sample of young men. Its second aim was to explore whether changes in work situation during the crisis and experiencing COVID-19 symptoms were associated with SES, level of education and working in contact with potentially infected people. The third aim was to investigate whether changes in participants’ work situation during the COVID-19 crisis were associated with its psychological impact (depression, perceived stress, sleep quality, psychological trauma, fear and isolation). Its fourth aim was to investigate whether psychological impact was associated with working in contact with potentially infected people and pre-crisis SES and level of education, even after adjusting for differences in experiencing COVID-19 symptoms and changes in work situation.

## Method

### Sample

This work was based on the Cohort Study on Substance-Use Risk Factors (C-SURF) and used data from survey waves shortly before and during the COVID-crisis. Participants had originally been contacted during the mandatory recruitment procedures testing young Swiss men’s fitness for military service, although data collection was kept independent of the army.

Participants were enrolled when they were approximately 19 years old in the years 2010–2012 at three of the six national military recruitment centres (in Lausanne, Windisch and Mels), together covering 21 of Switzerland’s 26 cantons. The study itself was conducted independent of the army, and participation did not depend on whether they served in the army or not. The Human Research Ethics Committee of the Canton of Vaud approved the research protocols for the parent C-SURF study and the COVID study (protocol 15/07 (PB_2018–00296). Between April 2019 and 14 February 2020, 4407 participants replied to the C-SURF study’s fourth wave questionnaire (hereafter the pre-COVID wave). On 13 May 2020, the pre-COVID wave participants were invited by e-mail and SMS to participate in the COVID study. Data collection for the COVID study was done online using LimeSurvey software [31] and ended on 8 June. A total of 2548 (57.8%) participants provided their informed consent to participate in the COVID study, and 2415 filled out at least the first section about their experiences of COVID-19 symptoms and their personal situation. Participants with missing values on predictor variables (about 3%) were excluded from the sample, resulting in a final study sample size of 2345.

### Measures

The questionnaire for the pre-COVID wave is available at https://www.c-surf.ch/en/30.html and for the COVID-19 wave questionnaire we adapted questions from this earlier wave of our cohort study where possible and added COVID-19 specific questions as described below.

#### The COVID-19 crisis’ psychological impact (outcome variables)

*Psychological distress during COVID-19 without mentioning it as a cause (measured before and during COVID-19)*. These measurements were asked about, in the same format, in both the pre-COVID and COVID-19 study questionnaires, and COVID-19 was not explicitly mentioned as a potential causal agent. Symptoms of major depression in the past two weeks were measured using the 12-item Major Depression Inventory (WHO–MDI) [32, 33], which was recoded into 10 items, forming a score ranging from 0–50. Perceived stress in the last month was measured using the 4-item short version of the Perceived Stress Scale [34], with items ranging from 0 (“never”) to 6 (“very often”). Sleep quality in the last month was measured using one question from the Pittsburgh Sleep Quality Index [35] with four response options ranging from 0 (“very bad”) to 3 (“very good”).

*Psychological distress due to COVID-19 (only measured during COVID-19)*. These measurements assigned distress to COVID-19 by using formulations such as, “due to COVID, I experienced…”. Perceived psychological trauma in the last seven days due to COVID-19 was measured using the 22-item Impact of Event Scale (IES), with three subscales measuring intrusion (e.g. “I had dreams about it”), avoidance (e.g. “I tried not to talk about it”) and hyperarousal (“I felt irritable and angry”) [36]. Response options ranged from 0 (“not at all”) to 4 (“extremely”), and the sum of the total scale ranged from 0–88. A cut-off of 24 was used as proxy for “at least some symptoms of psychological trauma” [37], and a cut-off of 33 was used for “probable psychological trauma” [38].

Fear due to COVID-19 since the beginning of the COVID measures was measured using 7 items asking about the degree to which participants were afraid of negative consequences due to the COVID-19 crisis. Questions covering fears for oneself, for others and financial fears were adapted from de Quervain, Aerni [39]. Response options ranged from 0 (“not at all”) to 4 (“extremely”).

Feelings of isolation since the beginning of the COVID-19 measures were measured using three questions asking how often participants felt isolated. Response options ranged from 0 (“never”) to 3 (“very often”). The questions were adapted from UCLouvain [40].

#### Predictors of psychological impact from the COVID study

Changes in work situation due to the COVID-19 crisis were based on questions asking participants whether their employment status changed due to the COVID-19 crisis since the beginning of the crisis (no change, job loss, partial unemployment, losing money as self-employed), whether their workload increased or decreased, and whether they were called up to their military or civil protection unit to assist in the COVID-19 crisis. Participants were also asked how many hours a week they worked from home and in total since the beginning of the crisis. Hours worked from home were divided by total hours worked and recoded as not working from home and working from home 1%–49%, from 50%–89% and from 90%–100%. Two questions were also asked to assess whether participants worked in contact with people potentially infected with COVID-19: they were asked whether they work as a healthcare professional and whether they worked at a workplace with regular contact with other people (for example food store or public transportation). This was based on participants’ individual perception of being at risk rather than objective criterion. Response options were “no”, “yes”, and “I am almost never / sometimes / often in contact with potentially infected people”. Responses were recoded to dichotomise between being a worker regularly in contact with potentially infected people (“yes”, “often” and “sometimes” coded as 1; “yes”, “almost never” and “no” coded as 0). Personal experience of COVID-19 symptoms since the beginning of the crisis was gauged using one question (with responses ranging from “no symptoms”, “symptoms without having been tested”, “tested negative” and “tested positive”). They were also asked whether other members of their household or entourage (e.g. family members or friends outside of the household) had experienced COVID-19 symptoms since the beginning of the crisis.

#### Predictors of psychological impact from the pre-COVID study

SES was assessed using two variables: relative financial status and difficulty paying bills. Relative financial status was assessed by asking how well-off participants considered themselves (adapted from [41]), with answers recoded to “below average”, “average” and “above average”. Difficulty paying bills was assessed using one question adapted from the Swiss Federal Statistical Office [42] asking whether participants had sufficient income to pay their usual bills/spendings at the end of the month. Their answers were recoded as “easy or very easy”, “fairly easy” and “rather difficult or difficult” to pay bills. The highest level of education attained was ascertained using one question in the pre-COVID survey wave, and answers were recoded into ISCED codes [43]. Current work situation before crisis was measured with one question asking about their current professional situation, and those that replied with “unemployment” were considered as having experienced unemployment before the crisis. This question was adapted from the Swiss Health Survey [44].

### Statistical analysis

Descriptive statistics were calculated to describe how the COVID-19 situation affected the work situation of our sample of young men (aim 1). Multinomial regressions were used to test whether SES, education and working in direct contact with potentially infected people were associated with experiencing symptoms of COVID-19 and changes in employment status (aim 2). Linear regression models were used to test associations between psychological impact and predictor variables from the COVID wave survey (changes in work situation during the crisis; aim 3), working in contact with potentially infected people, and pre-crisis SES, unemployment and level of education (aim 4). Outcomes were z-standardized (mean = 0, SD = 1) before the analysis to enable better comparisons of coefficient effect sizes across outcomes. Thus, the coefficients correspond to the differences in outcome, in standard deviations, for an increase of one unit in the predictor variable. All regressions were adjusted for participants’ age and linguistic region (German- vs French-speaking). Models for depression, perceived stress and sleep quality were also adjusted with regard to their baseline level in the pre-COVID wave survey. Regressions for the associations between psychological impact and SES, unemployment, level of education and working in direct contact with potentially infected people were adjusted for changes in employment status, workload, working from home and symptoms of COVID-19 experienced (by participants and their entourage). To test for differences between non-respondents and respondents to the COVID wave in the variables measured in the pre-COVID wave, we conducted t-tests for continuous variables and multinomial regressions for categorical variables as supplementary analysis to gauge for selective dropout. Relative bias [45] was also calculated to estimate the relative differences between participants and the total invited sample. Overall, compared to non-participants, those participating in the COVID-wave were significantly more often from the French-speaking region of Switzerland (58.0% versus 53.4%; relative bias -3.8%), reported significantly less often having difficulty paying their bills (26.1% vs. 32.3%; relative bias 10.1%) and had lower stress levels (4.89 vs. 5.46; relative bias 5.2%; S3 Table).

## Results

### Changes in employment status (aims 1 and 2)

Descriptive statistics are presented in Table 1, and participants’ perceived psychological impact of the COVID-19 crisis are presented in Table 2. About one fifth (21%) of the sample either lost their job (3.5%, compared to 1.9% being unemployed before the crisis), became partially unemployed (14.5%) or lost money as self-employed workers (3.1%), and more than 40% worked predominantly from home during the crisis (Table 1). While the large majority did not report a high psychological impact due to the crisis, 4.7% reported some symptoms of psychological trauma 3.5% reported symptoms consistent with psychological trauma, thus a considerable psychological impact (Table 2). Table 3 describes differences in experiences of COVID-19 symptoms and changes in employment status according to the highest level of education achieved, SES and working in contact with potentially infected people. A below-average financial status was associated with significantly higher odds of job loss, whereas an above-average financial status was associated with significantly lower odds of partial unemployment and having lost money as a self-employed worker due to COVID-19. Difficulty paying bills was associated with higher odds of job loss, partial unemployment and having lost money as a self-employed worker, although the latter two options were only significant for those who had replied “rather difficult or difficult”. Participants working in regular contact with potentially infected people had higher odds of having had COVID-19 symptoms. Those working in the healthcare sector also had lower odds of having seen changes in their employment status.

**Table 1:** Demographic characteristics of the sample (Total n = 2345).

	n	%
Age (mean/SD)	2345	29.07 (SD = 1.28)
**Linguistic region**		
French-speaking	1361	58.0
German-speaking	984	42.0
**Change in work situation due to COVID-19 measures**		
**Change in employment because of COVID-19**		
no change	1852	79.0
lost job	81	3.5
partially unemployed	339	14.5
self-employed and lost money	73	3.1
**Change in workload** ^a^		
decreased	646	27.5
no change	1066	45.5
increased	363	15.5
**Percentage working from home during COVID-19** ^a^		
90% to 100%	801	34.2
50% to 89%	200	8.5
1% to 49%	341	14.5
does not work from home	733	31.3
**Called up to military or civil protection unit (%yes)**	244	10.4
**Education**		
**Highest level of education (International Standard Classification of Education; ISCED)**		
compulsory schooling (ISCED 2; 9 years)	41	1.7
secondary school diploma (ISCED 34; 12–13 years)	221	9.4
apprenticeship (ISCED 35; 12–13 years)	944	40.3
bachelor’s degree (ISCED 6; 15 years)	612	26.1
master’s degree (ISCED 7; 17 years)	527	22.5
**Socioeconomic status before the crisis**		
**Relative financial status**		
below average	737	31.4
average	684	29.2
above average	924	39.4
**Difficulty paying usual bills**		
easy or very easy	971	41.4
fairly easy	762	32.5
rather difficult or difficult	612	26.1
**Unemployment before the crisis**		
Unemployment before the crisis (% yes)	45	1.9
**Working in regular contact with potentially infected people**		
**Job in healthcare sector in contact with patients (% yes)**	107	4.6
**Other job in contact with people (e.g. restaurant; % yes)**	538	22.9

Note

^a^ Total n does not include people not currently working.

**Table 2:** Descriptive statistics for psychological impact of the COVID-19 crisis.

Psychological distress during COVID-19 without mentioning it as a cause	mean	SD	%
**Major depression** score (n = 2228; range from 0 to 50)	7.60	7.79	7.0% (mild; 21+)
**Perceived stress** score (n = 2212; range from 0 to 16)	4.73	2.98	20.8% (≥ 8; sometimes on average)
**Sleep quality** (n = 2223; range from 0 to 3)	2.01	0.68	18.8% (rather bad or bad)
**Psychological distress due to COVID-19 mentioning it as a cause**			
**Psychological trauma** due to COVID-19 (n = 2240; sum of items, range 0 to 88)	7.98	10.23	4.7% some symptoms (24+) 3.5% probable trauma (33+)
**Fear** due to COVID-19 (n = 2260; range from 0 (not at all) to 4 (extremely)	1.02	0.68	10.6% at least moderate (2+)
**Isolation** due to COVID-19 (n = 2211; range from 0 (never) to 3 (very often)	0.65	0.64	6.0% at least often (2+)

**Table 3:** Results of multinomial regressions testing associations between experiences of COVID-19 symptoms and change in employment status, with socioeconomic status, highest level of education and working in contact with potentially infected people.

	Exposure to COVID-19				Change in employment status because of COVID-19			
	no symptoms (ref)	had symptoms but tested negative OR [95%CI]	had symptoms but was not tested OR [95%CI]	tested positive OR [95%CI]	no change (ref)	lost job OR [95%CI]	partial unemployment OR [95%CI]	self-employed and lost money OR [95%CI]
**Socioeconomic status before the crisis**								
**Relative financial status** (ref: average; n = 684)								
below average (n = 737)	ref.	1.34 [0.71, 2.51]	1.00 [0.74, 1.34]	7.50 [0.91, 61.57]	ref.	**5.56 [2.76, 11.18]**	1.00 [0.75, 1.34]	1.48 [0.88, 2.49]
above average (n = 924)	ref.	0.80 [0.41, 1.56]	1.00 [0.76, 1.33]	**9.58 [1.22, 75.10]**	ref.	1.19 [0.53, 2.69]	**0.65 [0.48, 0.86]**	**0.52 [0.28, 0.95]**
**Difficulty paying usual bills** (ref: easy or very easy; n = 971)								
fairly easy (n = 762)	ref.	0.95 [0.50, 1.81]	1.09 [0.83, 1.44]	0.69 [0.20, 2.40]	ref.	**3.15 [1.50, 6.64]**	1.18 [0.90, 1.56]	1.52 [0.83, 2.80]
rather difficult or difficult (n = 612)	ref.	1.53 [0.82, 2.82]	1.27 [0.95, 1.70]	1.82 [0.65, 5.10]	ref.	**6.46 [3.18, 13.16]**	**1.35 [1.01, 1.80]**	**3.20 [1.83, 5.62]**
**Highest level of education** (ref: apprenticeship (12–13 years; n = 944))								
compulsory schooling (9 years; n = 41)	ref.	**4.04 [1.29, 12.63]**	0.55 [0.19, 1.57]	2.24 [0.27, 18.59]	ref.	**7.40 [2.70, 20.32]**	1.52 [0.66, 3.50]	3.35 [1.19, 9.39]
secondary school (12–13 years; n = 221)	ref.	0.80 [0.27, 2.35]	0.83 [0.54, 1.29]	0.92 [0.20, 4.29]	ref.	1.64 [0.71, 3.75]	1.01 [0.69, 1.50]	1.09 [0.53, 2.23]
bachelor’s degree (15 years; n = 612)	ref.	1.55 [0.84, 2.88]	0.91 [0.67, 1.22]	0.51 [0.14, 1.89]	ref.	**1.79 [1.00, 3.20]**	**0.69 [0.51, 0.93]**	0.58 [0.32, 1.06]
master’s degree (17 years; n = 527)	ref.	0.84 [0.39, 1.81]	1.07 [0.80, 1.44]	0.74 [0.22, 2.44]	ref.	0.76 [0.36, 1.59]	**0.49 [0.35, 0.69]**	**0.36 [0.18, 0.72]**
**Working in contact with potentially infected people**								
**Job in healthcare sector in contact with patients** (ref: no; n = 1963)								
yes (n = 107)	ref.	**3.70 [1.68, 8.17]**	1.03 [0.58, 1.83]	**9.16 [3.09, 27.18]**	ref.	na	**0.38 [0.18, 0.83]**	na
**Other job in contact with people (e.g. restaurant)** (ref: no; n = 1534)								
yes (n = 538)	ref.	**2.61 [1.49, 4.58]**	**1.53 [1.17, 2.00]**	1.91 [0.69, 5.28]	ref.	1.18 [0.66, 2.11]	0.94 [0.71, 1.25]	1.34 [0.57, 3.13]

Note: 95%CI = 95% confidence interval of OR. na: no coefficient showed because of empty cells. Coefficients in bold are significant at the p < .05 level.

#### Associations with psychological impact

Associations between psychological impact and a change in work situation are reported in Table 4; associations between psychological impact and pre-crisis level of education, SES and working in contact with potentially infected people, adjusted for changes during the crisis, are reported in Table 5 (non-adjusted results are presented in S1 Table). For outcomes reported with no mention of COVID-19 as a cause, both before and during the crisis (depression, perceived stress and sleep quality), the analyses in Tables 4 and 5 were adjusted for baseline values, whereas S2 Table reports these associations without a baseline adjustment.

**Table 4:** Associations between psychological impact of the COVID-19 crisis with change in work situation, call up to military or civil protection unit and working from home.

	Psychological impact of the COVID-19 crisis					
	Psychological distress during COVID-19 without mentioning it as a cause *Measured during COVID-19 and adjusted for pre-COVID-19 levels*, *age and linguistic region*			Psychological distress due to COVID-19, mentioning it as a cause *Measured during COVID-19 only*, *and adjusted only for age and linguistic region*		
	Depression *b [95%CI]*	Perceived stress *b [95%CI]*	Sleep Quality *b [95%CI]*	Psychological trauma *b [95%CI]*	Fear *b [95%CI]*	Isolation *b [95%CI]*
**Change in work situation**						
**Change in employment status due to COVID-19** (ref: no change; n = 1852)						
lost job (n = 81)	0.09 [-0.11, 0.29]	**0.25 [0.05, 0.44]**	-0.03 [-0.23, 0.17]	**0.35 [0.12, 0.57]**	**0.61 [0.39, 0.83]**	**0.23 [0.01, 0.45]**
partial unemployment (n = 339)	-0.03 [-0.14, 0.07]	0.00 [-0.10, 0.11]	-0.01 [-0.11, 0.10]	0.10 [-0.02, 0.22]	**0.26 [0.15, 0.37]**	0.01 [-0.11, 0.12]
self-employed and lost money (n = 73)	**0.36 [0.14, 0.57]**	**0.38 [0.16, 0.59]**	-0.17 [-0.38, 0.05]	**0.41 [0.17, 0.64]**	**0.52 [0.29, 0.74]**	0.20 [-0.04, 0.43]
**Change in workload** (ref: no change; n = 1066)						
increase (n = 363)	**0.14 [0.03, 0.25]**	**0.12 [0.01, 0.23]**	**-0.19 [-0.30, -0.08]**	**0.17 [0.05, 0.29]**	**0.19 [0.08, 0.31]**	**0.15 [0.03, 0.27]**
decrease (n = 646)	0.08 [0.00, 0.17]	0.08 [-0.01, 0.17]	-0.02 [-0.11, 0.07]	**0.17 [0.07, 0.27]**	**0.18 [0.09, 0.28]**	0.07 [-0.03, 0.17]
**Called up to military or civil protection unit and working from home**						
**Called up to military or civil protection unit** (ref: no; n = 2101)						
yes (n = 244)	-0.05 [-0.17, 0.07]	0.11 [-0.01, 0.23]	-0.01 [-0.14, 0.11]	-0.01 [-0.15, 0.12]	0.11 [-0.03, 0.24]	0.03 [-0.10, 0.17]
**Percentage working from home** (ref: no work from home; n = 733)						
90% to 100% (n = 801)	**0.25 [0.16, 0.34]**	**0.16 [0.07, 0.25]**	-0.08 [-0.17, 0.02]	0.02 [-0.08, 0.12]	**0.14 [0.04, 0.24]**	**0.16 [0.06, 0.26]**
50% to 89% (n = 200)	0.10 [-0.04, 0.24]	0.03 [-0.11, 0.17]	-0.12 [-0.26, 0.02]	-0.11 [-0.27, 0.05]	-0.01 [-0.16, 0.14]	0.02 [-0.14, 0.17]
1% to 49% (n = 341)	0.05 [-0.07, 0.16]	0.04 [-0.08, 0.16]	-0.08 [-0.20, 0.03]	-0.02 [-0.15, 0.12]	0.04 [-0.09, 0.16]	-0.03 [-0.15, 0.10]

Note: Outcomes were z-standardized, and b represents differences in standard deviations with respect to the reference group. 95%CI = 95% confidence interval of b. All coefficients were adjusted for linguistic region and age. Coefficients in bold are significant at the p < .05 level.

**Table 5:** Associations between the psychological impact of the COVID-19 crisis and socioeconomic status, highest level of education, unemployment before the crisis and working in contact with potentially infected people; adjusted for experiences of COVID-19 symptoms and work situation during the COVID-19 crisis.

	Psychological impact of the COVID-19 crisis					
	Psychological distress during COVID-19 without mentioning it as a cause *Measured during COVID-19 and adjusted for pre-COVID-19 levels*, *age*, *linguistic region and participants’ experiences of the crisis* ^*a)*^			Psychological distress due to COVID-19, mentioning it as a cause *Measured during COVID-19 only and adjusted for age*, *linguistic region and participants’ experiences of the crisis* ^*a)*^		
	Depression *b [95%CI]*	Perceived stress *b [95%CI]*	Sleep Quality *b [95%CI]*	Psychological trauma *b [95%CI]*	Fear *b [95%CI]*	Isolation *b [95%CI]*
**Socioeconomic status before the crisis**						
**Relative financial status** (ref: average; n = 684)						
below average (n = 737)	**0.12 [0.03, 0.22]**	**0.15 [0.05, 0.25]**	-0.01 [-0.11, 0.08]	**0.15 [0.04, 0.26]**	**0.20 [0.10, 0.30]**	**0.19 [0.08, 0.29]**
above average (n = 924)	**0.10 [0.00, 0.19]**	0.00 [-0.09, 0.09]	0.02 [-0.07, 0.12]	-0.03 [-0.13, 0.07]	-0.07 [-0.17, 0.02]	0.08 [-0.02, 0.18]
**Difficulty paying usual bills** (ref: easy or very easy; n = 971)						
fairly easy (n = 762)	0.00 [-0.09, 0.08]	0.01 [-0.08, 0.09]	-0.05 [-0.14, 0.04]	**0.11 [0.02, 0.21]**	**0.14 [0.05, 0.23]**	0.04 [-0.05, 0.14]
rather difficult or difficult (n = 612)	0.01 [-0.09, 0.11]	**0.13 [0.03, 0.23]**	**-0.10 [-0.20, 0.00]**	**0.36 [0.26, 0.47]**	**0.35 [0.25, 0.45]**	**0.21 [0.11, 0.31]**
**Highest level of education** (ref: apprenticeship (12–13 years; n = 944))						
compulsory schooling (9 years; n = 41)	-0.12 [-0.41, 0.17]	0.04 [-0.25, 0.32]	0.05 [-0.24, 0.34]	-0.01 [-0.33, 0.31]	-0.01 [-0.31, 0.29]	-0.15 [-0.46, 0.16]
secondary school (12–13 years; n = 221)	**0.18 [0.04, 0.31]**	0.08 [-0.05, 0.21]	**-0.14 [-0.28, 0.00]**	0.09 [-0.06, 0.24]	0.00 [-0.14, 0.14]	**0.21 [0.06, 0.35]**
bachelor’s degree (15 years; n = 612)	0.09 [0.00, 0.19]	-0.03 [-0.13, 0.07]	-0.05 [-0.15, 0.05]	-0.10 [-0.20, 0.01]	0.09 [-0.01, 0.19]	0.10 [-0.01, 0.20]
master’s degree (17 years; n = 527)	**0.21 [0.11, 0.32]**	**0.11 [0.01, 0.22]**	-0.07 [-0.18, 0.04]	-0.01 [-0.12, 0.11]	0.04 [-0.07, 0.16]	**0.19 [0.08, 0.31]**
**Unemployment before the crisis** (ref: no = 2300)						
yes (n = 45)	0.13 [-0.15, 0.40]	0.08 [-0.20, 0.35]	0.13 [-0.15, 0.41]	**0.40 [0.09, 0.71]**	0.11 [-0.19, 0.40]	**0.43 [0.13, 0.73]**
**Working in contact with potentially infected people**						
**Job in healthcare sector in contact with patients** (ref: no; n = 1963)						
yes (n = 107)	0.01 [-0.16, 0.19]	-0.04 [-0.22, 0.14]	-0.01 [-0.20, 0.17]	-0.12 [-0.32, 0.08]	**-0.21 [-0.40, -0.02]**	0.00 [-0.19, 0.20]
**Other job in contact with people (e.g. restaurant)** (ref: no; n = 1534)						
yes (n = 538)	-0.06 [-0.15, 0.03]	-0.06 [-0.15, 0.03]	0.02 [-0.07, 0.12]	0.02 [-0.08, 0.12]	0.03 [-0.07, 0.12]	0.03 [-0.07, 0.13]

Note: Outcomes were z-standardized, and b represents differences in standard deviations with respect to the reference group. 95%CI = 95% confidence interval of b.

^a)^ participants’ experiences of the crisis was measured in form of experience of COVID-19 symptoms, COVID-19 symptoms in entourage, changes in employment status, change in workload, call up military or civil protection unit, percentage of work at home. Coefficients in bold are significant at the p < .05 level.

*Associations between psychological impact and a change in work situation during the crisis (aim 3)*. Having lost a job due to COVID-19 or having lost money as a self-employed worker were both associated with greater psychological impact in the form of depression (only self-employed workers who lost money), perceived stress, and levels of psychological trauma, fear and isolation (not significant for self-employed workers who lost money). Those in partial unemployment only showed significantly higher levels for fear (Table 4).

Changes in workload (increases or decreases) were associated with higher levels of fear, isolation (only significant for increases) and psychological trauma. An increase in workload was also associated with worse depression and perceived stress, as well as worse sleep quality. Working mostly from home (90%–100%) was associated with higher levels of depression, perceived stress, fear and isolation.

*Associations between psychological impact and working in contact with potentially infected people*, *pre-crisis socioeconomic status and level of education (aim 4)*. On most scales, a perceived below-average relative financial status and difficulty paying bills were associated with worse effects than were a perceived average income and ease paying bills (Table 5): greater levels of depression (only significant for relative financial status), perceived stress, psychological trauma, fear and isolation, as well as worse sleep quality (only significant for difficulty paying bills). An above average financial status was associated with significantly higher levels of depression after adjustment for baseline (0.10 [0.00, 0.19]; Table 5), but this was not significant without baseline adjustment (b = 0.05 [-0.05, 0.15]; S2 Table), meaning that they did not have significantly higher levels of depression during COVID-19, but started on a lower level before the crisis than those with an average financial situation. Participants who were unemployed when they filled out the pre-COVID questionnaire showed higher levels on all indicators of psychological impact, but this was only significant for psychological trauma and isolation, possibly due to the relative low n (only 45 participants). For participants outside the healthcare sector working in regular contact with potentially infected people, there were no significant differences in psychological impact, and those working in the healthcare sector in regular contact with potentially infected patients only reported significantly less fear, compared to participants not working in regular contact with potentially infected people. Baseline-adjusted (Tables 4, 5) coefficients for depression, perceived stress and sleep quality were somewhat lower than in the unadjusted analysis (S1 Table).

## Discussion

The present study focuses on associations between the psychological impact of the COVID-19 crisis and socioeconomic status (SES) and work situation. In addition to the pandemic’s direct health effects on our sample of young Swiss men, the economic impacts of the crisis and of the measures to counter it were considerable. The COVID-19 situation affected the employment status of more than a fifth, who either lost their job or, more frequently, became partially unemployed or lost money as self-employed. About 10% were called up to their military or civil protection unit to assist public services with the COVID-19 situation. More than 40% of the sample reported having to work predominantly from home.

### Associations between psychological impact and working from home and employment status

Being able to work from home could be seen as a privilege for the better educated, white-collar worker, as well as being less stressful as they are protected against infection with the virus at the workplace, or on their way there [1]. However, the present study’s results showed that those benefits might be outweighed by other factors in our sample: working mostly (90%+) from home was associated with higher levels of isolation, fear, depression and perceived stress. These results are somewhat in contrast with a study from Austria that found that the possibility to work from home was a protective factor for the psychological impact of the COVID-19 crisis [9]. A study in Switzerland also found that working from home was not associated with life satisfaction, but was associated with higher reduction in levels of perceived stress, albeit this was no longer significant after adjustment for socioeconomic resources [25]. However, these studies did not investigate the degree of working from home, and working partially (1% to 89%) from home was also not significantly associated with psychological impact in our sample and this may be preferable when the situation allows it. Further research may be needed to investigate which degree of working from home is best tolerated by which specific population subgroups. Nevertheless, should measures to contain a pandemic make full-time work from home compulsory, they should, where possible, be accompanied by measures to help reduce the psychological effects of changes in the work environment, fewer social contacts and a disrupted rhythm of life.

Changes in employment status (job loss or losing money as a self-employed worker) due to COVID-19 were also associated with higher levels of depression, fear and psychological trauma. However, partial unemployment was only associated with fear, but not with psychological trauma or depression. There are two potential explanations for this: firstly, partial unemployment is usually associated with fewer financial consequences and is meant as a temporary measure, thus causing fewer psychological distress; secondly, partial unemployment is rarely a measure aimed at a specific employee but is rather a business level decision, less likely to be taken personally, and linked to external economic circumstances that will be less associated with psychological distress. This is consistent with earlier findings that unemployment due to workplace downsizing or closures is associated with fewer negative health outcomes than being dismissed from a stable workplace [46]. The present study thus highlighted the advantages of partial unemployment over full layoffs as regards employee well-being and mental health.

### Workers in direct contact with potentially infected people

While large parts of the population could stay at home during the crisis, this was not possible for everyone and a part of the workforce had to continue to work in direct contact with people and were therefore exposed to a higher risk of infections. Of our participants, 22.9% indicated to have been in regular contact with potentially infected people outside the healthcare sector and 4.6% within the healthcare sector. Of note, this was based on the participant’s individual perception of risk rather than an objective assessment of infection risk (about which relatively few was known in the beginning of the crisis). Participants who perceived to be working in direct contact with potentially infected people outside the healthcare sector were somewhat more likely to have experienced symptoms of COVID-19 (but not significantly to have been tested positive) compared to participants without regular contact with potentially infected people, but were not differently affected in their employment status, and they showed few differences in psychological impact compared to those working in a setting with less contacts. In contrast, those working in healthcare in contact with potentially infected patients were more likely to have had COVID-19 symptoms and been tested positive. This was consistent with findings from the 2002 SARS epidemic, when 20% of all cases were among healthcare workers [47], and findings from the current epidemic in the USA and UK [48]. However, this may be partly due to the greater availability of tests in healthcare settings at the beginning of the crisis [48]. On the other hand, healthcare workers were very unlikely to be partially or fully unemployed and, overall, they showed few differences in psychological impact from other professions. This does not mean that our sample’s healthcare workers felt no psychological effects of the COVID-19 crisis, just that these were no greater than in other professions. Maybe relative financial security was a more important overall factor than the adaptations required of healthcare workers during the first wave of the crisis. Findings regarding healthcare workers’ mental health during the COVID-19 pandemic have been somewhat inconsistent, with many studies reporting a higher psychological impact among healthcare workers [19], while others found no such associations [27]. Some studies of the 2002 SARS outbreak did report long-term mental health consequences among health workers exposed to that virus [26, 49, 50]. Thus, even though our sample’s healthcare workers did not fare worse during the pandemic’s first wave, it will be important to observe whether there are any delayed consequences related to being continuously overworked, especially during the crisis’ second wave. The second wave was ongoing at the time of writing (November 2020), and case and hospitalisation rates far exceeded those of Switzerland’s first wave [51].

### Associations between psychological impact and socioeconomic status and education

Lower SES pre-crisis was associated with job loss, becoming partially unemployed and losing money due to COVID-19 as self-employed. Lower SES was also associated with higher psychological impact from the crisis in the form of depression, perceived stress, lower sleep quality, fear, isolation and psychological trauma. Besides being more likely to lose their job (e.g. those working in restaurants, shops, etc.), those with lower SES may have had fewer savings for periods of lower or no income and have had more problems living on unemployment benefits, which, in Switzerland, are from 70%–80% of one’s insured income. They may thus have suffered worse psychological effects due to financial distress. Our analyses were adjusted for experiencing COVID-19 symptoms and changes in work situation, and coefficients in the adjusted analyses were moderately lower than in the unadjusted analyses. Thus, these differences are only partially the effect of having experienced more changes in work situation; they also reflect an overall worse reaction to the COVID-19 crisis among people with a lower SES. Furthermore, coefficients for depression and perceived stress were still significant despite being adjusted for their pre-COVID wave values, albeit somewhat lower than in the non-adjusted analysis. Thus, these associations were also not only due to pre-existing differences in the variables: the mental health of participants with a low SES deteriorated relatively more during the COVID-19 crisis than among those with average or higher SES. Experiences from earlier crises (e.g. natural disasters) and initial results from the ongoing COVID-19 crisis have shown that crises reinforce and exacerbate disparities [1, 11, 12, 18, 19]. Compared to the countries where these results came from, Switzerland has a very generous social security safety net and universal healthcare access. It is thus particularly noteworthy that, in Switzerland too, differences in SES were nonetheless also associated with psychological impact of the crisis. However, levels of major depression were also higher in those with an above average relative financial situation compared to those with an average financial situation, a finding that was also found by Daly, Sutin [23] who reported lower mental health in those in the top tertile of income compared to those in the lowest tertile. In our study this difference was only significant after baseline adjustment, meaning that they showed lower levels of major depression before the crisis and showed a relative increase (compared to those with an average financial situation), showing the importance of longitudinal data to differentiate between effects of the crisis and pre-existing differences. With regard to level of education, a higher level of education was associated with a higher psychological impact in our study, which was consistent with similar findings from the United Kingdom [23], and a possible explanation may be that those with higher education were looking for more health related information which may have impacted their mental health negatively [23]. Our findings for education may seem somewhat at odds with our results for SES (relative financial status and difficulty to pay bills). However, SES and education were not very strongly correlated in our sample (results not reported) of young Swiss men, of around thirty: those with a higher level of education may still have been in the education system (e.g. doctoral studies, internships, etc.) and thus not necessarily have much money available and potentially even less stable jobs than those with an apprentice’s qualification. Overall, while our study and many studies report a higher psychological impact among those with lower SES [1, 11, 12, 18, 19, 25], it would seem that there is not a simple linear relationship between all indicators of SES (income, education, relative financial situation) [21, 23] and the psychological impact of the COVID-19 crisis. More research will be needed about in which subpopulations lower SES is a risk factor for a higher psychological impact due to the COVID-19 crisis and the mechanism linking SES to psychological impact.

### Limitations

Our participants were exclusively young Swiss men, and results may not be entirely generalizable to broader population groups. All the measures were self-reported, thus—especially the measurements of mental health—are not as precise as a clinical assessment. Furthermore, working in regular contact with potentially infected people was exclusively based on participants individual perception of risk. While this individual perception is certainly relevant with respect to psychological impact, no information was available about their objective risk exposure. Pre-COVID-19 questionnaires were returned across a 9-month period and COVID-19 questionnaires over a 4-week period, therefore the time between the two waves was variable over the entire sample. While we measured outcomes before and during the crisis and attributed these changes to the crisis, we cannot exclude that these changes would have had happened without the crisis, weakening the potential for causal interferences from our result. Furthermore, respondents to the COVID-19 questionnaire differed from non-respondents on several variables measured before the crisis. Finally, the present study took place early in the pandemic and thus no long-term effects could be assessed.

## Conclusion

The COVID-19 crisis had a high impact on the work situation and the psychological well-being of the young Swiss men in our sample. Those who lost their job or had to work mostly from home reported higher psychological impact due to the COVID-19 crisis, and such changes in work situation should be accompanied by supportive measures to reduce their psychological impact. Moreover, subgroups with a lower pre-crisis SES reported a higher psychological impact due to the COVID-19 crisis, thus, the crisis revealed and amplified pre-existing psychological frailties. Policy makers should ensure that measures taken to contain pandemics do not disproportionally affect already vulnerable groups, for example by offering the possibility of partial unemployment to sectors most affected by the crisis, which are often also the sectors with lower salaries. Where disproportional burdens on vulnerable groups cannot be avoided, accompanying measures should be taken to lessen the impact of the crisis on these subgroups. For example, providing easily accessible psychological and emergency financial support for those at a greater risk of experiencing psychological distress due to crisis may be an important element of crisis management. Such measures could also help to prevent crises from further augmenting disparities in mental health [11, 13].

## Supporting information

S1 Table Unadjusted ^a^ analysis from Table 4: Associations between the psychological impact of the COVID-19 crisis and socioeconomic status, highest level of education and working in contact with potentially infected people. Note: Outcomes were z-standardized, and b represents differences in standard deviations with respect to the reference group. 95%CI = 95% confidence interval of b. a In contrast to Table 4, this analysis was only adjusted for language and age, respectively baseline for consequences measured before and after COVID-19, but not for changes during the COVID-19 crisis (experience of COVID-19 symptoms, COVID-19 symptoms in entourage, changes in employment status, change in workload, call up to military or civil protection unit, percentage of work at home). Coefficients in bold are significant at the p < .05 level.
(PDF)

S2 Table Associations between depression, stress and sleep quality during the COVID-19 crisis without baseline adjustments for change in employment status, change in workload, call up to military or civil protection unit, percentage of work at home, socioeconomic status, highest level of education and working in contact with potentially infected people. Note: Outcomes were z-standardized, and b represents differences in standard deviations with respect to the reference group. 95%CI = 95% confidence interval of b. Analysis only adjusted for linguistic region and age. Coefficients in bold are significant at the p < .05 level.
(PDF)

S3 Table Differences in the variables measured before COVID-19 in non-respondents and respondents in the COVID-19 wave. Note: t-tests were used to test difference in continuous variables and multinomial regressions for categorical variables (with % indicators). Non-respondents did either not reply at all or only partially and were therefore excluded from the study. The relative bias was calculated by the following formula: value total ((n not included/n included in study)* (value not included/value included in study))/value total sample).
(PDF)
