# Supplementary material for: The psychological impact of the COVID-19 crisis is higher among young Swiss men with a lower socioeconomic status: Evidence from a cohort study
Source: PLoS One. 2021 Jul 29;16(7):e0255050. doi: 10.1371/journal.pone.0255050 (PMC8320994; doi:10.1371/journal.pone.0255050)
Supplement: S1 Table — Note: Outcomes were z-standardized, and b represents differences in standard deviations with respect to the reference group. 95%CI = 95% confidence interval of b. a In contrast to Table 4, this analysis was only adjusted for language and age, respectively baseline for consequences measured before and after COVID-19, but not for changes during the COVID-19 crisis (experience of COVID-19 symptoms, COVID-19 symptoms in entourage, changes in employment status, change in workload, call up to military or civil protection unit, percentage of work at home). Coefficients in bold are significant at the p < .05 level. (PDF) [file pone.0255050.s001.pdf]

S1 Table. **Unadjusted** [a] analysis from Table 4: Associations between the psychological impact of the COVID-19 crisis and socioeconomic status, highest level of education and working in contact with potentially infected people

	Psychological impact of the COVID-19 crisis					
	Psychological distress during COVID-19 without mentioning it as a cause *Measured during COVID-19 and adjusted for pre-COVID-19 levels*			Psychological distress due to COVID-19, mentioning it as a cause *Measured during COVID-19 only*		
	Depression *b [95%CI]*	Perceived stress *b [95%CI]*	Sleep Quality *b [95%CI]*	Psychological trauma *b [95%CI]*	Fear *b [95%CI]*	Isolation *b [95%CI]*
**Socioeconomic status**						
**Relative financial status** (ref: average; n = 684)						
below average (n = 737)	**0.17 [0.07, 0.26]**	**0.21 [0.11, 0.30]**	-0.03 [-0.13, 0.06]	**0.19 [0.08, 0.29]**	**0.26 [0.16, 0.37]**	**0.23 [0.12, 0.33]**
above average (n = 924)	**0.11 [0.02, 0.20]**	0.01 [-0.08, 0.10]	0.01 [-0.08, 0.10]	-0.05 [-0.15, 0.06]	-0.08 [-0.18, 0.02]	0.09 [-0.01, 0.19]
**Difficulty paying usual bills** (ref: easy or very easy; n = 971)						
fairly easy (n = 762)	0.00 [-0.09, 0.08]	0.02 [-0.07, 0.10]	-0.05 [-0.14, 0.04]	**0.12 [0.03, 0.22]**	**0.15 [0.06, 0.25]**	0.04 [-0.06, 0.13]
rather difficult or difficult (n = 612)	0.02 [-0.08, 0.12]	**0.17 [0.08, 0.27]**	**-0.11 [-0.20, -0.01]**	**0.41 [0.31, 0.52]**	**0.42 [0.32, 0.52]**	**0.23 [0.13, 0.34]**
**Highest level of education** (ref: apprenticeship (12–13 years); n = 944)						
compulsory schooling (9 years; n = 41)	-0.06 [-0.34, 0.23]	0.18 [-0.11, 0.47]	0.02 [-0.27, 0.31]	0.20 [-0.12, 0.53]	0.29 [-0.03, 0.60]	0.03 [-0.29, 0.34]
secondary school (12–13 years; n = 221)	**0.24 [0.10, 0.37]**	**0.16 [0.02, 0.29]**	**-0.16 [-0.30, -0.03]**	0.15 [0.00, 0.29]	0.10 [-0.05, 0.24]	**0.26 [0.11, 0.40]**
bachelor's degree (15 years; n = 612)	**0.15 [0.06, 0.24]**	0.00 [-0.09, 0.10]	-0.06 [-0.16, 0.03]	-0.10 [-0.20, 0.00]	0.09 [-0.01, 0.19]	**0.11 [0.01, 0.22]**
master's degree (17 years; n = 527)	**0.26 [0.17, 0.36]**	**0.13 [0.03, 0.22]**	-0.07 [-0.16, 0.03]	-0.06 [-0.17, 0.05]	-0.02 [-0.13, 0.09]	**0.19 [0.08, 0.29]**
**Working in contact with potentially infected people**						
**Job in healthcare sector in contact with patients** (ref: no; n = 1963)						
yes (n = 107)	-0.02 [-0.19, 0.15]	-0.08 [-0.25, 0.09]	-0.02 [-0.20, 0.15]	-0.10 [-0.30, 0.09]	-0.19 [-0.38, 0.00]	0.02 [-0.17, 0.21]
**Other job in contact with people (e.g. restaurant)** (ref: no; n = 1534)						
yes (n = 538)	**-0.12 [-0.21, -0.04]**	-0.08 [-0.17, 0.00]	0.03 [-0.06, 0.12]	0.06 [-0.04, 0.15]	0.04 [-0.05, 0.14]	0.00 [-0.10, 0.10]

Note: Outcomes were z-standardized, and b represents differences in standard deviations with respect to the reference group. 95%CI = 95% confidence interval of b. [a] In contrast to Table 4, this analysis was only adjusted for language and age, respectively baseline for consequences measured before and after COVID-19, but not for changes during the COVID-19 crisis (experience of COVID-19 symptoms, COVID-19 symptoms in entourage, changes in employment status, change in workload, call up to military or civil protection unit, percentage of work at home). Coefficients in bold are significant at the p <.05 level.
