# Supplementary material for: The psychological impact of the COVID-19 crisis is higher among young Swiss men with a lower socioeconomic status: Evidence from a cohort study
Source: PLoS One. 2021 Jul 29;16(7):e0255050. doi: 10.1371/journal.pone.0255050 (PMC8320994; doi:10.1371/journal.pone.0255050)
Supplement: S2 Table — Note: Outcomes were z-standardized, and b represents differences in standard deviations with respect to the reference group. 95%CI = 95% confidence interval of b. Analysis only adjusted for linguistic region and age. Coefficients in bold are significant at the p < .05 level. (PDF) [file pone.0255050.s002.pdf]

S2 Table. Associations between depression, stress and sleep quality during the COVID-19 crisis **without baseline adjustments** for change in employment status, change in workload, call up to military or civil protection unit, percentage of work at home, socioeconomic status, highest level of education and working in contact with potentially infected people.

	Depression without baseline adjustment b [95%CI]	Perceived stress without baseline adjustment b [95%CI]	Sleep quality without baseline adjustment b [95%CI]
**Change in employment status** (ref: not much changed; n = 1852)			
lost job (n = 81)	**0.34 [0.12, 0.56]**	**0.37 [0.15, 0.59]**	-0.10 [-0.32, 0.13]
partial unemployment (n = 339)	0.05 [-0.07, 0.16]	0.09 [-0.02, 0.21]	-0.06 [-0.18, 0.06]
self-employed and lost money (n = 73)	**0.43 [0.20, 0.67]**	**0.43 [0.20, 0.67]**	-0.22 [-0.46, 0.02]
**Change in workload** (ref: no change; n = 1066)			
does not work (n = 270)	**0.54 [0.41, 0.67]**	**0.59 [0.46, 0.73]**	**-0.22 [-0.35, -0.08]**
increase (n = 363)	**0.21 [0.09, 0.33]**	**0.14 [0.02, 0.26]**	**-0.30 [-0.42, -0.18]**
decrease (n = 646)	**0.15 [0.05, 0.24]**	**0.12 [0.03, 0.22]**	-0.07 [-0.17, 0.03]
**Call up to military or civil protection unit** (ref: no; n = 2101)			
yes (n=244)	0.05 [-0.09, 0.18]	**0.17 [0.04, 0.31]**	-0.07 [-0.21, 0.06]
**Percentage of work at home** (ref: no work from home; n = 733)			
does not work (n =2 70)	**0.56 [0.42, 0.70]**	**0.57 [0.43, 0.71]**	**-0.21 [-0.35, -0.07]**
90% to 100% (n = 801)	**0.24 [0.14, 0.34]**	**0.11 [0.01, 0.21]**	-0.10 [-0.20, 0.00]
50% to 89% (n = 200)	0.08 [-0.08, 0.23]	0.00 [-0.15, 0.16]	-0.13 [-0.29, 0.03]
1% to 49% (n = 341)	0.02 [-0.11, 0.15]	-0.03 [-0.16, 0.10]	-0.07 [-0.20, 0.06]
**Socioeconomic status**			
**Relative financial status** (ref: average; n = 684)			
below average (n = 737)	**0.33 [0.22, 0.44]**	**0.34 [0.24, 0.45]**	-0.09 [-0.20, 0.01]
above average (n = 924)	0.05 [-0.05, 0.15]	-0.04 [-0.14, 0.06]	0.05 [-0.05, 0.16]
**Difficulty paying usual bills** (ref: easy or very easy; n = 971)			
fairly easy (n = 762)	0.07 [-0.03, 0.17]	**0.12 [0.02, 0.21]**	-0.10 [-0.19, 0.00]
rather difficult or difficult (n = 612)	**0.35 [0.24, 0.45]**	**0.46 [0.36, 0.56]**	**-0.30 [-0.41, -0.20]**
**Highest level of education** (ref: apprenticeship (12–13 years; n = 944))			
compulsory schooling (9 years; n = 41)	**0.38 [0.06, 0.70]**	**0.51 [0.19, 0.83]**	-0.12 [-0.44, 0.20]
secondary school (12–13 years; n = 221)	**0.34 [0.19, 0.49]**	**0.18 [0.03, 0.32]**	**-0.23 [-0.37, -0.08]**
bachelor's degree (15 years; n = 612)	**0.13 [0.03, 0.23]**	-0.09 [-0.19, 0.01]	-0.06 [-0.17, 0.04]
master's degree (17 years; n = 527)	**0.24 [0.13, 0.35]**	0.04 [-0.07, 0.15]	-0.05 [-0.16, 0.06]
**Working in contact with potentially infected people**			
**Job in healthcare sector in contact with patients** (ref: no; n = 1963)			
yes (n =107)	-0.03 [-0.21, 0.16]	-0.10 [-0.29, 0.08]	-0.03 [-0.23, 0.16]
**Other job with contact with people (e.g. restaurant)** (ref: no; n = 1534)			
yes (n = 538)	**-0.11 [-0.20, -0.01]**	-0.06 [-0.15, 0.04]	0.01 [-0.09, 0.11]

Note: Outcomes were z-standardized, and b represents differences in standard deviations with respect to the reference group. 95%CI = 95% confidence interval of b. Analysis only adjusted for linguistic region and age. Coefficients in bold are significant at the p <.05 level.
