# Supplementary material for: The psychological impact of the COVID-19 crisis is higher among young Swiss men with a lower socioeconomic status: Evidence from a cohort study
Source: PLoS One. 2021 Jul 29;16(7):e0255050. doi: 10.1371/journal.pone.0255050 (PMC8320994; doi:10.1371/journal.pone.0255050)
Supplement: S3 Table — Note: t-tests were used to test difference in continuous variables and multinomial regressions for categorical variables (with % indicators). Non-respondents did either not reply at all or only partially and were therefore excluded from the study. The relative bias was calculated by the following formula: value total ((n not included/n included in study)* (value not included/value included in study))/value total sample). (PDF) [file pone.0255050.s003.pdf]

S3 Table. Differences in the variables measured before COVID-19 in non-respondents and respondents in the COVID-19 wave

| | Not included in study | Included in study | t-value or OR (multinomial regression) | p-value | relative bias |
|---|---|---|---|---|---|
| n | 2062 | 2345 | | | |
| Age | 28.17 | 28.21 | -0.97 | .330 | -0.1% |
| **Linguistic region** | | | | | |
| French-speaking | 53.4% | 58.0% | 1.205 | .002 | -3.8% |
| German-speaking | 46.6% | 42.0% | ref. | | |
| **Highest level of education (International Standard Classification of Education; ISCED)** | | | | | |
| compulsory schooling (ISCED 2; 9 years) | 2.3% | 1.7% | 0.802 | .324 | 12.1% |
| secondary school diploma (ISCED 34; 12–13 years) | 11.6% | 9.4% | 0.841 | .105 | 9.9% |
| apprenticeship (ISCED 35; 12–13 years) | 41.8% | 40.3% | ref. | | |
| bachelor’s degree (ISCED 6; 15 years) | 24.4% | 26.1% | 0.184 | 1.109 | -3.1% |
| master’s degree (ISCED 7; 17 years) | 19.9% | 22.5% | 1.173 | .055 | -5.7% |
| **Relative financial status** | | | | | |
| below average | 32.8% | 31.4% | 0.968 | .670 | 2.0% |
| average | 29.5% | 29.2% | ref. | | |
| above average | 37.7% | 39.4% | 1.057 | .453 | -2.1% |
| **Difficulty paying usual bills** | | | | | |
| easy or very easy | 35.5% | 41.4% | ref. | | |
| fairly easy | 32.2% | 32.5% | 0.864 | .043 | -0.4% |
| rather difficult or difficult | 32.3% | 26.1% | 0.691 | .000 | 10.1% |
| **Mental health** | | | | | |
| Major depression score | 9.54 | 9.10 | 1.86 | .063 | 2.2% |
| Perceived stress score | 5.46 | 4.89 | 6.51 | .000 | 5.2% |
| Sleep quality | 2.98 | 3.00 | -0.84 | .402 | -0.3% |

Note: t-tests were used to test difference in continuous variables and multinomial regressions for categorical variables (with % indicators). Non-respondents did either not reply at all or only partially and were therefore excluded from the study. The relative bias was calculated by the following formula: value total ((n not included/n included in study)* (value not included/value included in study))/value total sample).
